# Doxycycline vs. macrolides in combination with a β-lactam antibiotic for the treatment of community-acquired pneumonia in inpatients

**DOI:** 10.1186/s40001-022-00912-8

**Published:** 2022-12-08

**Authors:** Raghad K. Aldhahri, Shahad G. Gabb, Ohoud A. Shoaib, Reem M. Almadani, Khalid Eljaaly, Abrar K. Thabit

**Affiliations:** Pharmacy Practice Department, Faculty of Pharmacy, King Abdulaziz University, 7027 Abdullah Al-Sulaiman Rd, Jeddah, 22254-2265 Saudi Arabia

**Keywords:** Community-acquired pneumonia, Doxycycline, Macrolides, Azithromycin, Clarithromycin

## Abstract

**Background:**

Hospitalized patients with non-severe community-acquired pneumonia (CAP) are treated with a β-lactam plus either a macrolide or doxycycline. Limited data exist on the effectiveness of the latter combination. Therefore, we aimed to compare the combination of doxycycline vs. macrolide when either is combined with a β-lactam from effectiveness and safety perspectives.

**Methods:**

This was a retrospective cohort study in CAP inpatients between December 2013 and November 2020. Patients were divided into BL-D (β-lactam plus doxycycline) and BL-M (β-lactam plus a macrolide [azithromycin or clarithromycin]) groups. The primary endpoint was time to clinical stability. Secondary endpoints included length of stay (LOS) and in-hospital mortality.

**Results:**

Of 197 patients included, 57 were in the BL-D arm and 140 were in the BL-M arm. Patients were similar at baseline, except for the presence of leukocytosis, risk factors for drug resistance, and duration of therapy (*P* < 0.05 for all comparisons). No difference in clinical cure rate was observed (94.7% vs. 91.4%; *P* = 0.43). Time to clinical stability and LOS were similar in both groups at 4 (*P* = 0.82) and 7 days (*P* = 0.62), respectively. While only three patients died, only one (from the BL-M group) was due to sepsis. Liver enzymes elevation was more prominent in the BL-M group (21.4% vs. 5.3%; *P* = 0.01). A subgroup analysis showed shorter time to clinical stability with clarithromycin but higher cure rates with azithromycin.

**Conclusions:**

Data on doxycycline use with a β-lactam are scarce. Our study showed that such regimen was comparable in effectiveness to regimens involving macrolides with a better safety profile.

## Background

Community-acquired pneumonia (CAP) can range in severity from mild, which is treated as an outpatient to severe necessitating admission to an intensive care unit (ICU) [1]. The clinical presentation of CAP includes respiratory symptoms, such as cough, sputum, dyspnea, chest pain, fever, and malaise, as well as infiltrates detected on chest X-ray [2]. The Infectious Disease Society Of America (IDSA) and the American Thoracic Society (ATS) have recently updated their guidelines on the diagnosis and management of CAP, where they recommended starting the empiric therapy with either a β-lactam (cefotaxime, ceftriaxone, ampicillin) plus a macrolide or a respiratory fluoroquinolone [3]. A secondary suggested alternative to the macrolide in the first regimen would be doxycycline in the presence of a contraindication to macrolides [3].

Doxycycline is a tetracycline antibiotic that is active against a wide range of CAP pathogens including Gram-positive organisms such as *Streptococcus pneumoniae* and *Staphylococcus aureus* (including community-acquired methicillin-resistant species), Gram-negative organisms such as *Klebsiella* spp., *Escherichia coli*, and *Haemophilus influenzae*, and atypical bacteria. The bacteriostatic action of doxycycline is intended to impair bacterial cell growth by binding allosterically to the 30S ribosomal subunit; therefore, inhibiting protein synthesis. The most common adverse effect associated with doxycycline includes gastrointestinal upset, particularly esophagitis, photosensitivity, headache, and temporary discoloration of teeth [4].

Azithromycin, clarithromycin, and erythromycin belong to the macrolides group of antibiotics. They are active against *S. pneumoniae* and some strains of *H. influenzae*. Macrolides are bacteriostatic and act by attaching to the bacterial 50S portion of ribosomes, hence hindering protein synthesis. They are generally tolerable; nonetheless, the most important adverse effect of macrolides is QTc interval prolongation, which requires monitoring of the electrocardiogram, especially in high-risk patients with underlying cardiovascular problems or those concomitantly receiving drugs carrying the same risk [5].

A retrospective cohort study in hospitalized adults with CAP evaluated a regimen consisting of ceftriaxone plus doxycycline in comparison with other suitable empiric antibiotic regimens. The study found that the combination of ceftriaxone plus doxycycline was more effective with a mortality benefit than the other regimens [6]. Another retrospective study based on data from a large prospective study in 885 adults with CAP found no difference between the combination of a β-lactam plus a macrolide and that of a β-lactam plus doxycycline in terms of time to clinical stability, mortality, and hospital length of stay (LOS) [7].

Due to the limited head-to-head comparison studies of the two-drug combinations in terms of clinical efficacy and safety, the latest IDSA/ATS guidelines for CAP management recommended that more research is conducted in this area. Therefore, we aimed to evaluate the effectiveness and safety of doxycycline vs. that of macrolide when either is combined with a β-lactam antibiotic in inpatients with CAP.

## Methods

### Study design and patients

We conducted retrospective cohort study of CAP patients admitted to King Abdulaziz University Hospital, Jeddah, Saudi Arabia between December 2013 and November 2020. Patients were divided into two treatment groups. The first group involved patients who received a β-lactam plus doxycycline (BL-D), whereas the second treatment group included patients who received a β-lactam plus a macrolide (BL-M). Approval of the study protocol was obtained from the Research Committee of the Biomedical Ethics Unit of the Faculty of Medicine at King Abdulaziz University, Jeddah, Saudi Arabia (Reference No. 459–20).

Eligible patients were adults (≥ 18 years) who were diagnosed with CAP (based on clinical symptoms, such as fever, elevated white blood cells count, cough, shortness of breath, and/or chest radiographic evidence of pulmonary infiltrate), admitted to an inpatient medical ward, and received a regimen comprised of BL-D or BL-M (the macrolides were either azithromycin or clarithromycin,) for at least 5 days. Exclusion criteria were pregnancy, switched therapy from a macrolide to doxycycline or vice versa, discharged or died within 48 h of diagnosis, had respiratory comorbidities (bronchial asthma, cystic fibrosis, bronchiectasis, or chronic obstructive pulmonary disease), active tuberculosis, a positive polymerase chain reaction (PCR) test for coronavirus disease 2019 (COVID-19), had cancer, or admitted to the ICU prior to therapy.

### Endpoints

The primary endpoint was time to clinical stability, defined as the resolution of clinical signs and symptoms of infection. Clinical failure was defined as the persistence of symptoms requiring a change of antibiotic therapy, admission to the ICU for mechanical ventilation or hemodynamic instability, or death presumed to be due to CAP. Secondary endpoints included LOS, ICU admission, 30 day readmission, and in-hospital mortality.

### Statistical analysis

Medians [interquartile range, IQR] were compared using Mann–Whitney U test. Chi-square or Fisher’s exact test was utilized to compare categorical variables. Kaplan–Meier curve was used to graph time to clinical stability, and a log-rank test was done to compare the differences. Cox regression was done to estimate the association of treatment group with the primary endpoint. An a priori* P* value of < 0.05 was used to determine statistical significance. SPSS version 24.0 software (SPSS, Inc., Chicago, Illinois, USA) was used for analysis. An estimated difference of 20% in the primary endpoint with an alpha error probability of 0.05 and 80% power were used to calculate the sample size of 197 patients based on results from previous studies [6, 7].

## Results

A total of 197 patients were eligible for the study, 57 patients in the BL-D group vs. 140 patients in the BL-M group. Of note, seven of the excluded patients were started on a macrolide and then switched to doxycycline due to QTc prolongation. Due to the prolonged antibacterial effect of macrolides (given their long half-life), which may extend to days after discontinuation, patients who underwent this switch were excluded from the analysis. ‏In general, patients in the two treatment groups were similar in their baseline characteristics, except for the percentage of patients who had risk factors for drug-resistant *S. pneumoniae* (DRSP), the count of these factors, the percentage of patients with leukocytosis, C-reactive protein, serum level, and the duration of therapy (Table 1).

**Table 1:** Patients and treatment characteristics

Characteristic	BL-D (n = 57)	BL-M (n = 140)	*P* value
Baseline characteristics			
Age (years)	62 [50.5–73]	56 [41.25–71.75]	0.078
Male	32 (56.1)	71 (50.7)	0.489
Presence of risk factors for DRSP	52 (91.2)	104 (74.3)	0.008
Number of risk factors for DRSP	3 [2, 3]	2 [0–3]	0.045
Risk factors for DRSP			0.071
Comorbidity^a^	52 (91.23)	92 (65.71)	
Diabetes mellites	38 (66.67)	65 (46.43)	
Immunosuppression	0 (0)	13 (9.29)	
Received antibiotic therapy within 3 months	3 (5.26)	4 (2.86)	
Age ≥ 65 years	25 (43.86)	42 (30)	
MRSA or *Pseudomonas* infection in the past year	1 (1.8)	1 (0.7)	0.509
Hospitalization or IV antibiotics within 3 months	6 (10.5)	12 (8.6)	0.666
Respiratory culture ordered	13 (22.8)	47 (33.6)	0.137
Chest infiltration	44 (78.6)	95 (73.6)	0.476
Baseline SpO_2_ (%)	93 [90.5–96]	94 [87–97]	0.745
Presence of Leukocytosis	23 (40.4)	83 (59.3)	0.016
Presence of fever	24 (42.1)	39 (27.9)	0.052
Baseline C-reactive protein, mg/L^b^	21.05 [7.1–63.7]	48.1 [20.5–136.5]	0.024
Treatment Characteristics			
Primary β-Lactam			0.284
Ceftriaxone	57 (100)	134 (95.7)	
Cefuroxime	0 (0)	4 (2.9)	
Amoxicillin	0 (0)	2 (1.4)	
Switched to another β-Lactam^c^			0.299
Cefuroxime	6 (85.7)	24 (57.1)	
Amoxicillin	0 (0)	14 (33.3)	
Cefazolin	0 (0)	1 (2.4)	
Ceftazidime	1 (14.3)	3 (7.1)	
Changed β-Lactam for discharge	7 (12.3)	42 (30)	0.009
DOT (days)	9 [7–13.5]	7 [6–10]	0.003

Data are presented as *n* (%) or median [IQR]

*BL-D* β-Lactam + doxycycline, *BL-M* β-Lactam + macrolide, *DOT* duration of therapy, *DRSP* drug resistance *Streptococcus pneumoniae*‏, *IQR* interquartile range, *SpO*_*2*_ oxygen saturation, *WBC* white blood cells, *MRSA* methicillin resistant *staphylococcus aureus*

^a^Comorbidity indicates a medical history of heart, lung, liver, and/or kidney disease

^b^Data available from 45 patients (24 in the BL-D group and 21 in the BL-M group)

^c^These are data of a subset of patients who were switched to another β-lactam during the course of therapy

The number of patients for whom a respiratory culture was ordered was a total of 60, 13 and 47 in the BL-D and BL-M groups, respectively. Only 28 of these cultures (46.7%) showed microbial growth. Most of these grown cultures (22/28; 78.6%) grew either normal flora (such as *Staphylococcus epidermidis* or *Corynebacterium* spp.) or yeast. One patient in the BL-D arm had methicillin-resistant *S. aureus* (MRSA), whereas the remaining five patients in the BL-M arm had *S. viridans* group*, K. pneumoniae, H. influenzae, H. parainfluenzae,* or *S. aureus.* Ceftriaxone was the most commonly used β-lactam in both groups (100% vs. 95.7%; *P* = 0.28).

**Fig. 1:**
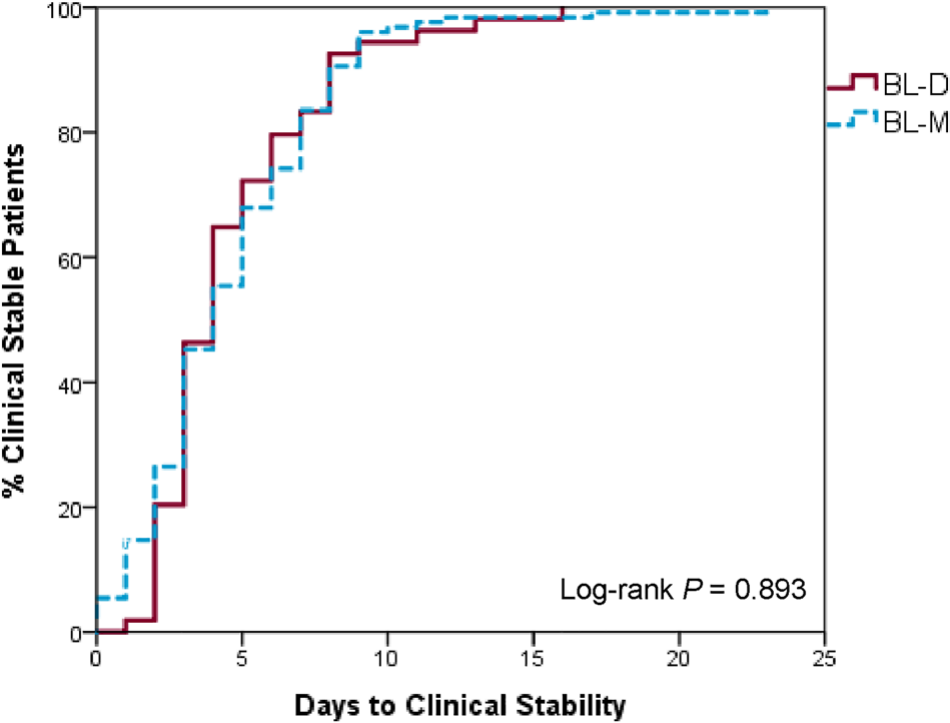
Time to clinical stability in the BL-D and BL-M groups. *BL-D* β-Lactam + doxycycline, *BL-M* β-Lactam + macrolide

Table 2 presents the clinical outcomes of patients, where there was no significant difference between the two groups in all primary and secondary outcomes. Leukocytosis, fever, and oxygen saturation were improved from baseline in all patients. Figure 1 shows the Kaplan–Meier curve of the time to clinical stability, where the log-rank test found no difference between the two groups (*P* = 0.893). Cox regression showed no association of treatment group with time to clinical stability (HR 1.18, 95% CI 0.79–1.75). In-hospital mortality was very low in both study arms. The reasons for mortality were intracranial hemorrhage of one patient in the BL-D group and underlying sepsis (*n* = 1), myocarditis (*n* = 1), and severe heart failure (*n* = 1) of the three deceased patients in the BL-M group. When liver enzymes were evaluated, significantly more patients in the BL-M group had elevation from baseline compared with the BL-D group (21.4% vs. 5.3%; *P* = 0.006). No other adverse events were reported in both groups.

**Table 2:** Patients outcomes

Outcome	BL-D (*n* = 57)	BL-M (*n* = 140)	*P* value
Time to clinical stability (days)	4 [3–6]	4 [2–7]	0.822
Clinical cure	54 (94.7)	128 (91.4)	0.427
LOS (days)	7 [5–9.5]	7 [5–10]	0.623
ICU admission	1 (1.8)	3 (2.1)	0.861
In-hospital mortality	1 (1.8)	2 (1.4)	0.866
30 day readmission	9 (15.8)	13 (9.3)	0.189
Resolved leukocytosis	9 (47.4)	34 (51.5)	0.750
Resolved fever	24 (100)	32 (94.1)	0.227
EOT SpO_2_, %	99.5 [99–100]	99 [98–100]	0.173
Elevation of liver enzymes	3 (5.3)	30 (21.4)	0.006

Results are presented as *n* (%) or median [IQR]

*BL-D* β-Lactam + doxycycline, *BL-M* β-Lactam + macrolide, *EOT* end of therapy, *ICU* intensive care unit, *LOS* length of stay, *SpO*_*2*_ oxygen saturation

A subgroup analysis of BL-M patients showed a higher cure rate with azithromycin vs. clarithromycin (97.3% vs. 84.8%; *P* = 0.009) with a shorter time to clinical stability (3 vs. 5 days; *P* < 0.0001) as shown on Table 3. A difference was also observed with liver enzymes elevation, which was more prominent with azithromycin (29.7% vs. 12.1%; *P* = 0.01). No difference was reported with the other outcomes.

**Table 3:** Subgroup analysis of patients who received macrolides

Outcome	BL-AZM (*n* = 74)	BL-CLR (*n* = 66)	*P* value
Time to clinical stability (days)	5 [3–7]	3 [1–4.75]	< 0.0001
Clinical cure	72 (97.3)	56 (84.8)	0.01
LOS (days)	6.5 [5–9.25]	8 [6–10.25]	0.02
ICU admission	0 (0)	3 (4.5)	0.06
In-hospital mortality	0 (0)	2 (3%)	0.13
30 day readmission	7 (9.5)	6 (9.1)	0.94
Elevation of liver enzymes	22 (29.7)	8 (12.1)	0.01

Results are presented as *n* (%) or median [IQR]

*BL-AZM* β-Lactam + azithromycin, *BL-CLR* β-Lactam + clarithromycin, *ICU* intensive care unit, *IQR* interquartile range, *LOS* length of stay

## Discussion

In this study, we showed that treatment with BL-D was comparable to treatment with BL-M in terms of clinical cure, time to clinical stability, as well as other clinical outcomes.

In a prospective study, Teh, et al. included 855 patients (178 in the BL-D group and 680 in the BL-M group), where they found lower rate of earlier clinical deterioration with BL-D than with BL-M; though, the difference did not achieve statistical significance (14.6% vs. 21.2%; *P* = 0.06) [7]. Our findings showed similar clinical cure rates in both groups. While the median time to clinical stability in the study by Teh, et al. was similar in both groups, the statistical significance of this variable could be attributed to the difference between the ranges rather than the difference between the medians (2 [0–7] vs. 2 [0–22] days; *P* = 0.006). Moreover, BL-D patients had shorter LOS but by only 1 day; thus, the statistical significance could be possibly attributed to the difference in the ranges (5 [0–26] vs. 6 [0–78] days; *P* = 0.001). The LOS of our patient cohort was close to that reported in this study, which had a median of 7 days (*P* = 0.623). Another study of 341 patients included 216 in the ceftriaxone plus doxycycline group and 125 in a group of patients who received appropriate comparative therapy (a β-lactam plus a macrolide or fluoroquinolone monotherapy) [6]. The study showed a significant lower inpatient and 30 day mortality in the ceftriaxone/doxycycline group vs. the comparator group (2.3% vs. 14.4% and 6% vs. 20%, respectively; *P* < 0.001 for both comparisons). Similar to our study, 30 day readmission rates were not different between these two groups; however, LOS was statistically different, although it was only 1 day shorter in the ceftriaxone/doxycycline group (3 vs. 4 days; OR = − 0.09, 95% CI − 0.25 to − 0.06). A prospective double-blinded clinical trial by Wiesner, et al. compared erythromycin to doxycycline in a total of 297 ambulatory patients with bronchitis or pneumonia [8]. In their study, both treatments were comparable and very effective with 96.6% and 97.2% success rates, respectively. Such results validate our findings of the high success rates reported with BL-D (94.7%) and BL-M (91.4%) regimens.

From an economic perspective, a regimen comprising doxycycline was significantly more cost-effective than a regimen with comparators, where hospitalization costs were lower by more than 20% in a randomized prospective trial of 87 patients, 43 in the doxycycline group vs. 44 in the control group (*P* = 0.04) [9]. Similarly, costs of antibiotic therapy were significantly lower in the doxycycline group (*P* < 0.001). That study also showed that doxycycline was associated with a shorter mean time to clinical stability and LOS compared with the control group (2.21 days vs. 3.84 days; *P* = 0.001 and 4.14 vs. 6.14 days; *P* = 0.04, respectively). A similar economic benefit with doxycycline in terms of lower hospitalization and treatment costs was also observed in another prospective double-blinded study comparing doxycycline to levofloxacin in 65 patients (*P* < 0.0001) with comparable clinical success rates (97.1% vs. 93.3%; *P* = 0.84) [10]. However, LOS was shorter in the doxycycline group (4 vs. 5.7 days; *P* < 0.001).

In our study, only 60 patients (30.5%) of the total cohort had respiratory cultures but only 28 (46.7%) showed microbial growth of either Gram-positive or Gram-negative bacteria despite the presence of respiratory symptoms at baselines that granted the inclusion of these patients in the study. The lack of bacterial growth could be pertinent to either the respiratory sample itself, initiation of antibiotic therapy before specimen collection, or that the causative organism was atypical bacteria, which require special growth conditions to appear in the cultures. Nonetheless, empiric coverage of atypical bacteria by either doxycycline or a macrolide is recommended by the IDSA/ATS guidelines, and a recent meta-analysis demonstrated lower clinical failure rates when atypical bacteria are targeted in empiric therapy [11]. Although the IDSA/ATS guidelines recommend the collection of microbiological specimens from hospitalized CAP patients to facilitate de-escalation of empiric therapy, no specific recommendations were made for culturing of atypical bacteria [3].

In terms of safety, seven patients who were originally allocated to the BL-D group were initiated on a macrolide with the β-lactam; however, this did not last long as they developed QT prolongation resulted in switching them to doxycycline. Those patients were not considered for the analysis and replaced with patients who continued the regimen with macrolides. Furthermore, more than one-fifth of the patients in the BL-M group experienced liver enzymes elevation from baseline. Although a recent meta-analysis has shown that clarithromycin has a better safety profile than erythromycin when used for the treatment of CAP [12], the risk of hepatotoxicity remains a concern with the former given the observations in our study, as well as previous reports [13]. Nonetheless, such adverse effect was more common with azithromycin than with clarithromycin in our study. While monitoring of liver enzymes would be prudent, it is assumed that the short CAP therapy course of 5 days might be insufficient to cause significant liver damage. However, monitoring would be recommended if the prolongation of the course was deemed clinically necessary in patients showing slow clinical improvement.

The subgroup analysis of azithromycin vs. clarithromycin showed a significant difference in both clinical cure and time to clinical stability. Conversely, a prospective, randomized, open-label study in 278 patients showed equivalent clinical cure rates in hospitalized patients with CAP who were treated with ceftriaxone plus azithromycin (114/135; 84.3%) vs. those treated with ceftriaxone plus clarithromycin or erythromycin (118/143; 82.7%), with a shorter mean LOS in the azithromycin group (10.7 vs. 12.6 days, respectively) [14]. Three randomized controlled trials also showed no difference in clinical cure rates between azithromycin and clarithromycin for non-severe pneumonia. O’Doherty et al. included 203 patients and found a similar satisfactory clinical response (94% vs. 95%; *P* = 0.518) [15]. Similarly, a clinical response of 100% was observed with both macrolides in a study on 70 patients [16]. Finally, a small study by Rizzato et al. of 40 patients found no significant difference in clinical cure (100% vs. 89%; *P* > 0.05) with a relatively shorter time to defervescence in the clarithromycin group (14 vs. 17 days; *P* > 0.05) [17].

Our study was limited by a few factors. The first was the small number of patients in the BL-D arm, which was attributed to the fact that doxycycline is commonly used for indications other than CAP, such as skin infections, vaginal infections, and brucellosis [18]. Second, some patients had insufficient data in their electronic medical records to determine clinical cure or failure; hence, they could not be included in the study. Third, the risk of bias and confounding factors cannot be excluded given the observational design of this study.

## Conclusions

Our study showed that a regimen comprised of BL-D was comparable to BL-M regimens in terms of effectiveness but with a favorable safety profile. Large randomized controlled trials are recommended to confirm these results.

## Data Availability

Data are available in the supplementary material.

## Abbreviations

CAP: Community-acquired pneumonia
COVID-19: Coronavirus disease 2019
ICU: Intensive care unit
IDSA/ATS: Infectious Diseases Society of America/American Thoracic Society
LOS: Length of stay
